# Challenging power and unearned privilege in physiotherapy: lessons from Africa

**DOI:** 10.3389/fresc.2023.1175531

**Published:** 2023-06-26

**Authors:** Stephanie Lurch, Saul Cobbing, Verusia Chetty, Stacy Maddocks

**Affiliations:** ^1^ School of Rehabilitation Science, Physiotherapy Program, McMaster University, Hamilton, ON, Canada; ^2^ Department of Physical Therapy, University of Toronto, Toronto, ON, Canada; ^3^ Department of Physiotherapy, University of KwaZulu-Natal, Durban, South Africa; ^4^ The Institute for Education Research, University Health Network, Toronto, ON, Canada; ^5^ Centre for Heart Lung Innovation, UBC and St. Paul’s Hospital, Vancouver, BC, Canada; ^6^ Department of Physical Therapy, University of British Columbia, Vancouver, BC, Canada

**Keywords:** Africa, physiotherapy, power, social justice, decoloniality

## Abstract

Power and unearned privilege in the profession of physiotherapy (PT) reside in the white, Western, English-speaking world. Globally, rehabilitation curricula and practices are derived primarily from European epistemologies. African philosophies, thinkers, writers and ways of healing are not practiced widely in healthcare throughout the globe. In this invited perspectives paper, we discuss the philosophies of Ubuntu and Seriti, and describe how these ways of thinking, knowing, and being challenge Western biomedical approaches to healthcare. We believe implementing these philosophies in the West will assist patients in attaining the health outcomes they seek. Further we call for Western professionals and researchers to stand in solidarity with their African counterparts in order to move towards a diversity of practitioners and practices that help to ensure better outcomes for all.

## Introduction

The roots of physical therapies may have begun centuries ago in Northern Africa (1) though in the United Kingdom, physiotherapy as a profession emerged in the late 1800s under somewhat dubious origins (2). While the profession has moved away from association with puritanical scandals, there are still some troubling aspects of physiotherapy that remain scandalous despite our more than one hundred years of movement. We argue in this paper that a) the centre of power and unearned privilege in physiotherapy still rests very squarely in the United Kingdom and its English-speaking “first-world” former colonies, and b) that we need more diversity of thought and people in order to move the profession forward.

Physiotherapy (3) and other health science curricula (4) are built on European epistemologies. In Africa, where English is often a second or third language and resources are often non-existent (euphemistically referred to as “constrained”), there exists past and present communities of distinguished physiotherapy practitioners, thinkers and researchers with ideas and practises that are not new but rather are yet to be explored by the Western world. That is, ideas, practices and philosophies that differ from those of the West have not permeated the profession of physiotherapy in any meaningful way. If, as someone from the global majority, you can speak the language of physiotherapy (both literally and metaphorically) you may receive a conditional pass into the exalted upper echelons of the trade, but to do so it helps to toe the line and not suggest too many changes to the status quo. Identity matters here. If you come from a distant unknown land you have more chance of being accepted in the Global North if you happen to be white, straight, able bodied, middle class, able to speak English, have been educated at an “acceptable” institution and have published in the requisite high-impact international (read: Western) journals. Therein lies the default PT identity (5).

Any discussion of power and privilege in physiotherapy has to acknowledge that representation in the profession (in most if not all countries) rarely reflects the population at large—white people make up a minority of the world’s population. People who are Black, identify as living with disability, as a member of the LGBTQ2S+ community and/or having come from rural, low socio-economic backgrounds and other historically marginalised groups are underrepresented in the physiotherapy student and qualified professional ranks. The interconnected nature of these social categorizations create intersecting systems of disadvantage that make it less likely for people from equity-denied communities to enter and thrive in our profession (5, 6). Intricately linked to this is the often-implied message that “Western is better”, in terms of what is taught, learned and practiced, where and what research is conducted, and who are deemed to be experts generally (7).

This invited perspectives paper highlights the need for a transformative anti-oppressive and anti-racist approach to rehabilitation care. This paradigm shift cannot be achieved without recognition of the work being done in Africa and by those of African descent. There are philosophies that are well-known in Africa and central to the way people live. These philosophies are not widely practiced in physiotherapy or the health professions but they may indeed hold the answers to some of the world’s most pressing issues. In this paper, we discuss African philosophies such as *Ubuntu* and *Seriti* and describe how these ways of thinking challenge Western biomedical approaches to health care. Further we call for Western professionals and researchers to stand in solidarity with their African PT counterparts in order to move towards a diversity of practitioners and practices that help to ensure better outcomes for all. We question the prevailing flow of information from the global minority to the global majority and will introduce readers to work published by African scholars in order to mobilize new ways to think, teach and practice.

## Positioning ourselves

The authors of this article are a team that bring a diversity of perspectives, experiences and identities. We are leaders, university faculty, post-doctoral students, clinicians, and community members. We each have our own history, language, memory and identities. Four hundred years and counting after the start of the colonial experiment, we are described thus: We are Black, white, and brown; male and female, Canadian and South African; of middle class origins and from humble beginnings; able bodied, heterosexual, Christian, agnostic and atheist. We are not all knowing, but we do have expertise credentialed by our scholarship and lived experience. Neither are we unbiased. What we all have in common is this: Our people come from Africa, we have all worked in Africa and we are all physiotherapists. We see not only the much-vaunted “potential” of the African continent but the significant lessons that the world can learn from Africa if only it resolves to listens. Not at some mystical time in the future. But right now.

There are, and have been for some time, a growing group of renowned physiotherapists from Africa who have been boldly addressing some of the most challenging questions in our profession and the broader fields of health sciences and health, including those arising and compounded by the COVID-19 pandemic. While it is important to declare our own positionality, we are intent on addressing this topic with as balanced a view as possible. Adopting a social justice lens (8), we insist that one cannot be selective on issues of power and privilege. While we extol the myriad benefits of embracing African approaches to health care, we are certainly not blind to some of the stark contradictions that exist on the continent. For example, no discussion of equity in our profession can ignore the highly problematic laws and attitudes that threaten people from the LGBTQ2S+ community in Africa, a situation that is itself compounded by the colonial project (9).

## Coloniality and systems of oppression

Analysis of systemic oppression must include a contextual and historical analysis of its root causes. Indeed, anti-oppression action can never be non-contextual or ahistorical (10). Neither can discussions about the root causes of oppression omit a critical analysis of power as illustrated by Quijano in his conceptual framing of the Colonial Matrix of Power and Western modernity (11). These serve to describe how hierarchical structures- racial, political, social, economic- ensured power resided with white Europeans and shaped the Western modern world (11, 12). This is the orientation we take here.

Quijano (11) notes one of the primary axes of global power was the social classification of the world’s population around race, which considered white Europeans as biologically superior and racialized non-Europeans as inferior. This became the criterion for separating people into ranks and roles- superior/inferior, dominated/dominator, white/Black. Over time, and in the course of the global expansion of colonial domination, a systematic racial distribution of labour was implemented. Europeans associated unpaid labour with inferior races/non Europeans and paid labour with superior races/Europeans. It is vital to note that race is a social construct, meaning it is made up. There is absolutely no biological basis for race (13).

Notably, domination over the new model of global power concentrated the control of knowledge and knowledge production in the West. Indeed, Europe was (and we posit remains) the centre of global epistemic power (4), and white European men remain “the canons of knowledge production in Westernised universities” (7). This model of power, based on coloniality, rendered non-European knowledge as inferior and primitive (12). The colonized had their forms of knowledge production, meaning making, symbolism, means of expression, and objective and subjective realities replaced by those of the colonizers resulting in the erasure of entire knowledge systems. Thus, “colonization did not only occur through physical seizing and displacement of peoples from land, but also through the colonization of minds” (7). This legacy of colonialism permeates contemporary society (including physiotherapy) and has negated the legitimacy of other forms of knowing, understanding and seeing.

## African philosophies of healthcare

*Ex Africa semper aliquid novum*. This is a Latin phrase translated as “out of Africa there is always something new”, attributed to Pliny the Elder in AD79. This is over 2000 years ago, and African ways of healing and philosophies of healthcare long predate this, but we feel it is important that readers are exposed to what to them may be “new” ways of thinking. Ramose (14) proposes that Western European understandings of Africa are based more on their historical interaction with African people, rather than a true appreciation of African people’s own self-understanding. Whether African people would have defined the much-contested term of “philosophy” in the same way as European scholars is a moot point. What is clear is that, across Africa, there is no doubt that there is a commonality in the way human interaction is practiced and understood, and this certainly extends to the domains of healthcare and healing. Perhaps the most well-known of these African philosophies is the concept of *Ubuntu*. Ubuntu is an isiXhosa/isiZulu word that can be defined as the idea that a person is a person through other people. It is known throughout Africa by other names—*Bomoto* (Democratic Republic of Congo), *Unhu* (Zimbabwe), *uMunthu* (Malawi), *Vumuntu* (Mozambique) and *Utu* (Kenya and Tanzania)—but the philosophy remains the same (15). Ubuntu describes personhood as something that is communally determined and does not emerge from an autonomous self (16). With regard to healthcare, this understanding of the self has a number of important ramifications. It should never be possible to treat a patient or teach students about healthcare without a very real focus on gaining a holistic understanding of all of the patients’ relationships with family members and the broader communities in which they live, work and play. By centreing the values of Ubuntu both structurally and institutionally and reinforcing the state of being human, Sambala et al. (15) argue that both health education and public health can be improved. The Northern Sotho/Setswana word *Seriti* describes the understanding that the essence of a human being is spiritual and can thus not be explained in material terms. It pertains more to ethical and metaphysical qualities that all people have. Not having an appreciation and understanding of a person’s Seriti, would render it impossible for them or their wider communities to achieve full dignity or health. Seriti and health are inextricably linked, one cannot fulfil one without the other (17).

Imafidion (16) describes how African philosophies of healthcare focus not only on healing the body and the mind, but in order for true health to be attained, one has to also focus on healing and building relationships and addressing any imbalances that may exist in an individual’s life. This resonates with the *Nguzo Saba* (Swahili for seven principles), which were conceptualised in 1966 by Dr Maulana Karenga, the professor of Black Studies at California State University, as a way of defining commonalities between North American peoples of the African diaspora (18). White and Estreet (19) describe how these principles can be applied to healthcare. For example Ujima (the principle of collective work and responsibility) dictates that family and political structures must not be ignored in any analysis of individual health or macro health systems. Gebremikael et al. (20) emphasise that the Nguzo Saba, Ubuntu and other African philosophies clearly understand health as being more than just the absence of disease, and far broader even than how the West conceptualises the social determinants of health. This brief summary of some of the African approaches to life and healthcare is not exhaustive. We strongly encourage practitioners and educators in our profession to read more, and engage directly with African people, listen more and learn. We are further aware that there are clear similarities between these philosophies and other Indigenous approaches to healthcare. We, the authors, are not in any way qualified to discuss North American Indigenous philosophies in depth. It appears clear, however, that there are similarities in African and Indigenous knowledge systems that can transform Western systems of healthcare practice and education. They both bring together opportunities for relationality, accountability, and creating safe spaces for healing and learning. Indeed, the Indigenous principles of Etuaptmumk (the Mi'kmaw word for Two-Eyed Seeing) have the potential to resolve the conflicts between Indigenous approaches to healthcare and the so-called scientific evidence-base that informs Western practice and teaching (21).

## Taking care to the people

Africa holds great profundity as a nation of people and offers valuable lessons on approaching change in healthcare. It is a continent that boasts a great diversity of people, who are often let down by lackluster healthcare systems where only the privileged thrive. Researchers have delved deep into these colonial-derived systems in trying to address the gaps that continue to prejudice the majority of people who access public healthcare services. A primary healthcare approach, predicated on the philosophy of Ubuntu, can improve access to care for people living in equity-denied communities, by providing health services in or near to the homes of people living with impairments and/or disabilities (22). A successful example of this is TAIBU (the Kiswahili word for “Be in good health”) Community Health Centre, a community driven organization offering culturally affirming primary care, health promotion and disease prevention to Black-identifying communities in Toronto, Canada. While this approach does show promise in Africa, gaps related to geopolitical challenges still hinder its successful implementation.

Another example of how current African approaches to health education and practice may prove beneficial to other regions across the globe is the growing implementation of decentralized approaches to clinical education. This approach sees student learning shifted from urban centres to rural communities where predominantly Black African people reside, and has proven useful to promote learning that is contextually-relevant for future health care practice (23). A study with physiotherapy students in KwaZulu-Natal, South Africa, demonstrated how this approach also increases the opportunities for students to be real advocates for equity-denied community members (24). Pillay and Kathard (25) described students’ exposure to longitudinal community engagement as a means to promote context-specific learning and decolonization of health education. This can also overcome some of the deficits in the content of health sciences curricula that often fail to integrate traditional medicine approaches in the management of African patients and where assessment tools have been developed within a colonial framework and for Eurocentric populations (26).

## The future of our profession reimagined

The future is an open temporal territory (10). The path we are suggesting is not an easy one, but challenging the accepted status quo in any profession in any era, has never been. Decolonial curricula open up, rather than close, possibilities for the expansion of the knowledge project, but prevailing systemic injustices are difficult to overcome (27). People of the privileged global minority, where the power of the profession undoubtedly resides, can choose to attend a few diversity workshops, and then return to doing the same work they have always done. Or they can do the real hard work, that necessarily involves ceding some of one's own power to the collective as in Ubunto and Seriti, not only because it seems to be the right and just thing to do but because this is the best for the future of our profession, communities and humanity. This will also require not only a political and economic resolve but a social resolve to consciously shift power and allow care to evolve using anti-colonial praxis. Lane et al. (28) argue that we need “brave spaces” rather than safe spaces in order to decolonize physiotherapy curricula and humanize healthcare. Standing in solidarity with equity-denied communities requires that people who hold power recognise their social location (i.e. the intersection of their identities) and use their unearned privilege to actively challenge the oppressive systems and practices that are still pervasive in the health professions (29). This includes making way for people from different backgrounds and with different experiences to their own, including African practitioners, thinkers and researchers. We do not want to romanticize Africa, and know full well that the continent has many challenges. But we have no doubt that ideas and practices related to African philosophies of healthcare can benefit practitioners and patients across the world, as it has in Africa for centuries. This approach will help researchers to ask the right questions, result in more diverse, socially-aware graduate cohorts and ensure better outcomes for all the people that we treat and advise.

## Data Availability

The original contributions presented in the study are included in the article, further inquiries can be directed to the corresponding author.
